# Long-term humoral immunogenicity, safety and protective efficacy of inactivated vaccine against reindeer rabies

**DOI:** 10.3389/fmicb.2022.988738

**Published:** 2022-09-08

**Authors:** Irina Matveeva, Olga Karpova, Nikolai Nikitin, Oleg Akilin, Vasiliy Yelnikov, Irina Litenkova, Roman Melnik, Nikolai Melnik, Karim Asimov, Aleksey Zaberezhny, Yriy Fyodorov, Evgeniya Markova

**Affiliations:** ^1^All-Russian Scientific Research and Technological Institute of Biological Industry, Biocombinat, Moscow Region, Russia; ^2^Department of Virology, Lomonosov Moscow State University, Moscow, Russia; ^3^Shchelkovo Biocombinat Federal State Enterprise, Biocombinat, Moscow Region, Russia

**Keywords:** rabies virus, vaccine strain, rabies vaccine, inactivated vaccine, reindeers, immunogenicity

## Abstract

The core element of the reindeer rabies eradication strategy is regular application of vaccines to obtain and uphold a vaccination coverage sufficient for the ceasing of rabies virus transmission. This article presents the results of reindeer humoral immunity intensity and duration study after the immunization with two form of inactivated rabies vaccines (adjuvanted liquid vaccine and non-adjuvanted lyophilized vaccine) based on the Shchelkovo-51 rabies virus strain. Efficiency of post-vaccine immunity was assessed by measuring the animal blood serum virus-neutralizing antibody level in a neutralization test. The study determined the efficient rabies vaccine injection dose as equal to 3 ml. A single dose of 3 ml of these vaccines induced stable production of specific neutralizing antibodies in reindeer as early as 7 day after administration, and by 30 days after immunization, it significantly exceeded the minimal threshold level accepted by OIE. Two doses of vaccines administration with an interval of 30 days are required to achieve a strong immunity with the rabies-specific virus-neutralizing antibody titer of more than 0.5 IU/ml for at least 2 years. Our data do not support the benefit of an adjuvanted vaccine for the prevention of rabies in reindeer.

## Introduction

Rabies is a viral disease of mammals and human, characterized by central nervous system impairment and inevitable mortality (WHO Publication, 2010; WHO, 2013; World Organization for Animal Health, 2016). Rabies virus is a typical member of the *Lyssavirus* genus of the *Rhabdoviridae* family. Its genome is a negative single-stranded non-segmented RNA, which encodes five structural proteins: nucleoprotein (N), phosphoprotein (P), matrix protein (M), glycoprotein (G) and RNA-dependent RNA-polymerase (L). Glycoprotein G is a sole antigen of the rabies virus able to induce virus-neutralizing antibodies (VNA) production (Johnson et al., 2010).

Rabies in terrestrial mammals is detected all across the globe with the exception of Australia and the Antarctic. Despite the successful eradication of the disease in several European countries, the epizootic status of many countries of Eurasia, Africa and the Americas remains worrying. In the Russian Federation, the situation with rabies also remains tense (Shulpin et al., 2018). Vaccination is deemed to be the most effective means of rabies prevention. Effective rabies vaccines are required to combat this infection, able to quickly induce a strong and long-term immunity after administration in the target animal.

A number of rabies vaccines are available in Russia for use in many animal species, but there is poor data on protective immunity of deer vaccinated in the field, although there are a few reports on the duration of immunity in deer (Sihvonen et al., 1993). Vaccines based on the Schelkovo-51 rabies strain have been successfully used for many years in Russia for the vaccination of pets and bovine animals. Here we research the level and duration of humoral immunity in reindeer following application of two forms of inactivated rabies vaccines based on the Schelkovo-51 strain, Liquid and Lyophilized. Effectiveness of the vaccines in reindeer was the subject of a several years of research (from 2012 to 2017); the studies were conducted at reindeer farms of the Republic of Sakha (Yakutia) where no outbreaks of infectious diseases were observed during the study. Unlike the Lyophilized Vaccine, the Liquid Vaccine additionally contains aluminum hydroxide as an adjuvant. Due to the fact that the air temperature in the Republic of Sakha (Yakutia) during the year varies from −35 to 20°C it is not possible to provide the proper temperature regime for storage and use of the Liquid vaccine under such conditions. A Lyophilized vaccine that can be stored and transported at any sub-zero temperature is a more appropriate form for use in such areas. Thus, here we also examined the contribution of the adjuvant in the Liquid vaccine to the formation of virus neutralizing antibody titers.

## Materials and methods

### Viruses, cells, and vaccines

Shchelkovo-51, RV-97, and Moscow 3,253 rabies virus strains were obtained from the collection of the All-Russian Scientific Research and Technological Institute of Biological Industry. Referential rabies virus strains, namely, the challenge virus standard (CVS)-11 brain strain, and the CVS-11 fixed culture strain, were provided by the OIE Reference Laboratory for Rabies of the French Agency for Food, Environmental and Occupational Health and Safety (ANSES) at Nancy, France.

The Shchelkovo-51 strain was propagated in suspension cell line BHK-21 (golden hamster kidney) and served as a seed virus for the development of the inactivated rabies vaccines against rabies.

Here we assessed the effectiveness of two vaccines: the Rabikov inactivated liquid cultural rabies vaccine (hereinafter referred to as “Liquid Vaccine”) and the inactivated lyophilized cultural rabies vaccine (hereinafter referred to as “Lyophilized Vaccine”). Both vaccines are made of the Shchelkovo-51 strain by the Shchelkovo Biocombinat Federal State Enterprise (Russia). A brief summary of the vaccine characteristics is available as Table 1.

**Table 1:** Liquid and lyophilized vaccines.

Core features	Liquid vaccine	Lyophilized vaccine
Commercial name	Rabikov	Inactivated lyophilized cultural rabies vaccine made of the Shchelkovo-51 strain
International non-propriety name	Inactivated liquid cultural rabies vaccine made of the Shchelkovo-51 strain (Rabikov)	Inactivated lyophilized cultural rabies vaccine made of the Shchelkovo-51 strain
Strain	Shchelkovo-51	Shchelkovo-51
Pharmaceutical form	Injection suspension	Injection suspension
Animal species	Bovine cattle, ruminants, horses	Bovine cattle, ruminants, horses, swine, dogs, cats.
Immunogenic activity	At least 1 IU	At least 1 IU
Inactivation	Beta-propiolactone with addition of 20% colloid aluminum oxide hydrate	Beta-propiolactone with addition of 33.3% of sucrose-peptone-gelatin stabilizer
Injection site	Subcutaneously	Subcutaneously, intramuscularly (swine).
Vaccination age	3 months or older	2 months or older
Storage conditions	From 2 to 8°С	From 2 to 8°С or any freezing temperature

Brief summary of product characteristics.

### Animals

Effectiveness of rabies vaccines was assessed using 3–6 month-old reindeer free from infectious diseases, bred at the Taba-Yana agricultural cooperative in the Ust-Yansk district of the Republic of Sakha (Yakutia).

This study was carried out in strict accordance with the 1986 Animals (Scientific Procedures) Act, in compliance with all the rules of Directive 2010/63/EU of the European Parliament and of the Council of the European Union of 22 September 2010 on the protection of animals used for scientific purposes (EU, 2010), Guide for the Care and Use of Laboratory Animals (National Research Council, 2011). The study on reindeers was approved by the Commission on Bioethics of All-Russian Scientific Research and Technological Institute of Biological Industry (protocol #2 dated 12.01.2012).

### Vaccination

**Figure 1:**
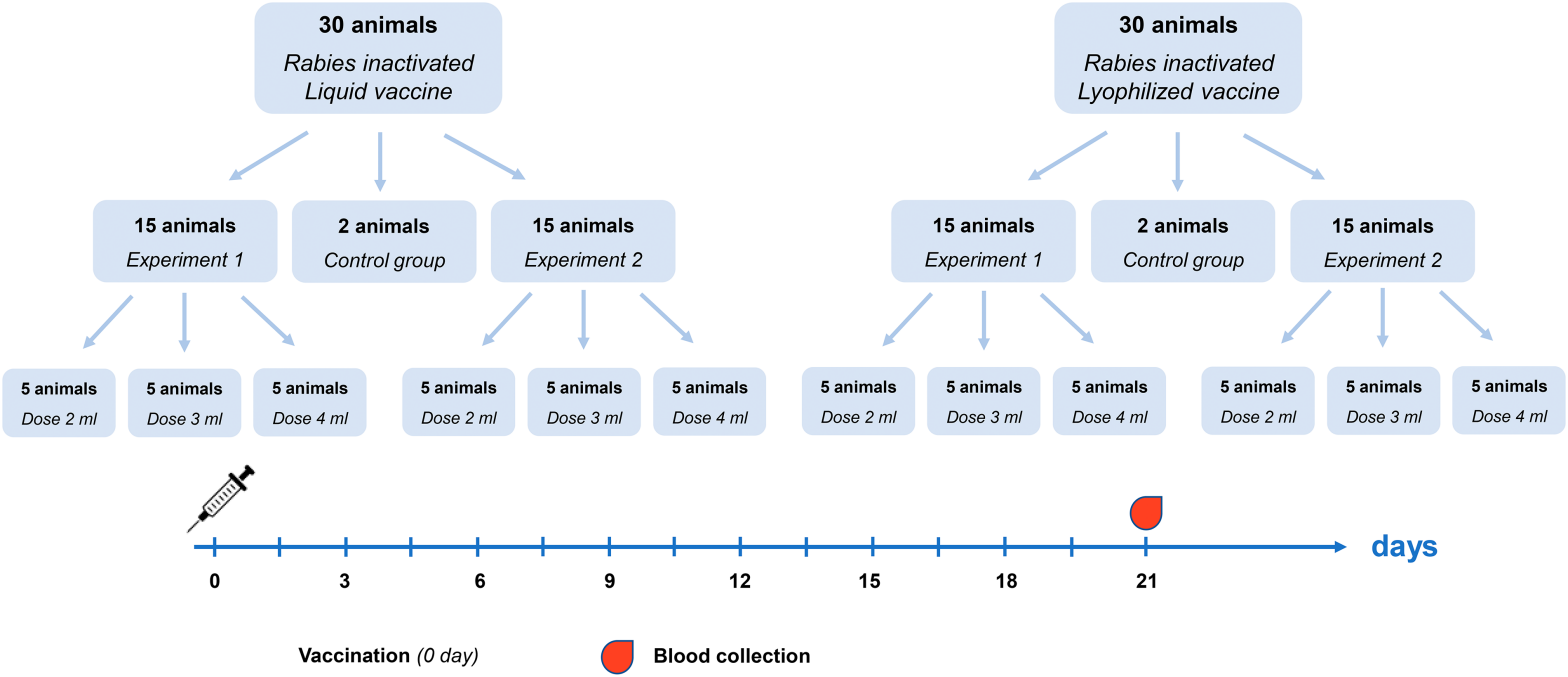
Vaccine dose determination experiment design.

For determination of effective vaccine dose 64 healthy previously unvaccinated reindeer were randomly divided into three groups: vaccination with Liquid vaccine (30 animals), vaccination with Lyophilized vaccine (30 animals) and a control group without vaccination (4 animals). The vaccines were administered on reindeer subcutaneously in doses of 2, 3, and 4 ml singly in the middle third of the neck. The experimental design used for vaccine dose determination is presented as Figure 1. Two replications of the experiment were conducted (Experiment 1 and Experiment 2). In each experiment, 15 animals were vaccinated with Liquid and Lyophilized vaccine (5 animals per each dose). Reindeer blood serum were collected 21 days after administration.

**Figure 2:**
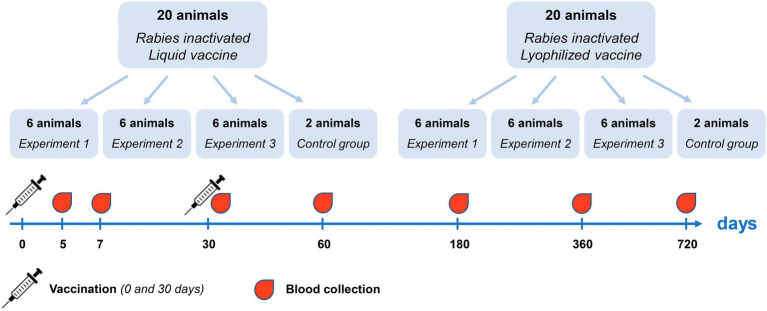
Experimental design for evaluation of immunity level following reindeer vaccination.

The reindeer immunity level was evaluated following double subcutaneous immunization, at 30 days interval, with Liquid and Lyophilized vaccines in doses equal to 3 ml. Within this research 40 healthy previously unvaccinated reindeer were randomly divided into three groups: vaccination with Liquid vaccine (20 animals), vaccination with Lyophilized vaccine (20 animals) and a control group without vaccination (4 animals). Three individual experiments were conducted (Experiment 1, Experiment 2 and Experiment 3). In each experiment, 6 animals were vaccinated with Liquid and Lyophilized vaccine. Reindeer blood serum was analyzed to determine titers of rabies virus-neutralizing antibodies at 5, 7, 30, 60 days and at 6, 12, and 24 months post first vaccination (Figure 2).

To assess vaccine safety, nine reindeer were used for each form of the vaccine, two independent experiments were conducted, and three animals were left unvaccinated and used as a control group. Both vaccines were administered in two-fold doses (6 ml) subcutaneously in the middle third of the neck. To accomplish this, contents of three vaccine flasks was combined, and 6 ml of resulting mixture were injected. Reindeer were observed for 21 days, recording their survival and physiological condition.

### Field trials of rabies vaccines in reindeer

Animals (total number of reindeer 4,359) were vaccinated twice (30 days interval) with Liquid and Lyophilized Vaccines in doses equal to 3 ml. 30 days after the second immunization 1,953 reindeer blood serum samples were collected to evaluate the efficiency of the vaccines (1,053 for the Liquid Vaccine and 900 for the Lyophilized Vaccine).

### Rabies virus neutralization assay

Deer serum samples were stored at −20°C and placed in a water bath at 56°C for 1 h to inactivate complement before use (Wager et al., 1968; Samuelsson and Herlitz, 2008; Krämer et al., 2009; Moore and Hanlon, 2010). The titers of virus-neutralizing antibodies in vaccinated reindeer blood serum and the duration of immunity were evaluated using the Fluorescent Antibody Virus Neutralization (FAVN) test (Cliquet and Wasniewski, 2015) and expressed in International Units per ml by comparing sample testing results with positive standard testing results (IU/ml; Kim et al., 2018), according to the OIE Manual of Diagnostic Tests and Vaccines for Terrestrial Animals (OIE, 2018). Every blood serum sample was examined thrice using specific diagnostic IgG immunoglobulins marked with fluorescein isothiocyanate (All-Russian Scientific Research and Technological Institute of Biological Industry) and international reference preparations, namely: CVS-11 (previously ATCC reference VR 959) strain, OIE lyophilized rabies standard, dog blood serum, capacity of 6.7 IU/ml and OIE negative standard, dog blood serum (ANSES, Nancy, France).

### Phylogeny analysis

Genotyping of Shchelkovo-51 vaccine virus strain samples was carried out *via* Sanger sequencing. The АВІ Prism 3,100 Genetic Analyzer (Applied Biosystems, United States) and BigDye Terminator ѵЗ.1 Cycle Sequencing Kit (Applied Biosystems, United States) were used in accordance with the manufacturer’s instructions to determine the nucleotide sequence of fragments.

Oligonucleotide primers were designed using the NCBI GenBank database and bioinformatics software, namely Vector NTI Advance 9.0 (PC), DNASTAR, BLAST.

The phylogenetic status of rabies strains in the *Rhabdoviridae* family was determined and phylogenetic trees designed *via* the MEGA 11 software using the Neighbor Joining method, 1,000 repeats of the Bootstrap Test of Phylogeny *p*-distance model and Maximum Parsimony method (Tamura et al., 2007). The homology level of studied sequences and sequences present in NCBI GenBank database was assessed using the BLAST software. Evolution divergence between sequences and the standard error was calculated using the MEGA 11 software. Sequences were aligned using the ClustalW algorithm.

### Statistical analysis

In reindeer immunity level evaluation after vaccination a Wilcoxon signed-rank test was used to confirm that the experimental groups exceeded the threshold of 0.5 IU/ml at day-7 and 24 months after the start of the study. Probability values (*p*-values) of less than 0.05 were considered to be significant. The graphs plotting and statistical analysis were carried out using GraphPadPrism 9.1.4 (GraphPad Software, La Jolla, San Diego, CA, United States).

## Results

The Glycoprotein G gene sequence was obtained for the Shchelkovo-51 strain (accession number ON181556). The GenBank registration number for this nucleotide sequence is: BankIt2569966 BSeq#1 ON181556.

**Figure 3:**
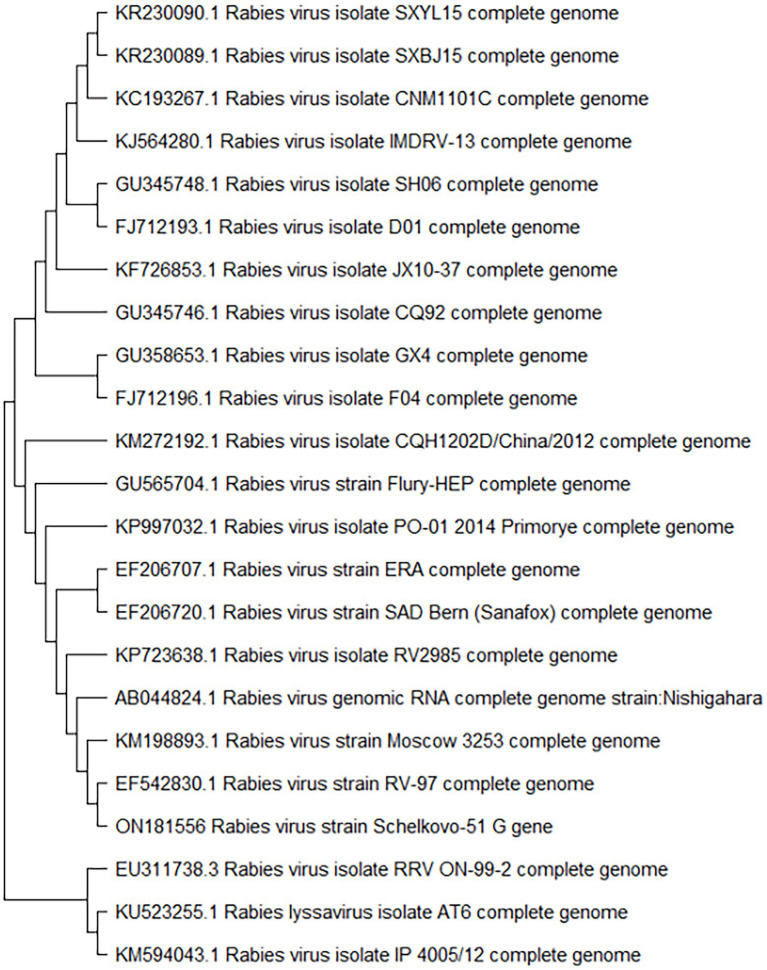
Phylogenetic analysis of glycoprotein G gene of rabies virus strains.

We analyzed the glycoprotein G gene sequence of the Shchelkovo-51 rabies virus strain, and evaluated the homology level of the gene in comparison to those of other rabies virus strains available in international databases, including vaccine strains RV-97, Moscow 3,253, Era, SAD, Nishigahara and Vnukovo-32. The results of the analysis demonstrated a high degree of homology (over 99%) with some Russian strains and homology in the region of 90% for other strains (Table 2). A phylogenetic tree diagram was built on the basis of similarity among glycoprotein G gene sequences in the rabies virus (Figure 3).

**Table 2:** Rabies virus isolates compared with Shchelkovo-51 strain in glycoprotein gene analysis.

Strain	GenBank accession no.	Collection date	Country	Nucleotide identities (%)
RV-97	EF542830.1	1997	Russia	99.8
Moscow 3253	KM198893.1	n/a	Russia	99.5
Nishigahara	AB044824.1	1915	Japan	95.3
Era	EF206707.1	1960	USA	92.9
Sad	EF206720.1	1935	USA	92.7
CVS-11	GQ918139.1	2010	China	89.0
Vnukovo-32	LT575363.1	1974	Russia	n/a^*^

n/a, not analyzed.

* The sequences presented here are related to different sites of the rabies virus genome, thus they cannot be aligned.

In order to control the quality of manufactured rabies vaccines as far as genetic stability of the glycoprotein G gene sequence is concerned, samples of cultural liquids of vaccine strain passages 2 and 16 were collected. Glycoprotein G gene sequences of the strain, reproduced in suspension cell line BHK-21 were 100% identical at passage 2 and 16 according to BLAST algorithm results (2,036 out of 2,036 bp identical; Supplementary Figure 1). The analysis results confirm the genetic stability of the main immunogenic viral protein, which is a crucial criterion for manufactured vaccines quality control.

### Rabies vaccines safety assessment

All vaccinated animals remained clinically healthy throughout the observation period, no physiological deviations were detected, body temperature, behavior and weight were normal for their age.

### Determination of effective vaccine dose

To determine the rabies vaccine dose, unvaccinated reindeer were selected, and their negative immunity status was confirmed *via* laboratory studies using the FAVN test (Johnson et al., 2010; Astray et al., 2017; blood serum rabies virus-neutralizing antibody titers amounted to less than 0.25 IU/ml). After that, reindeer were immunized one time in doses equal to 2, 3 and 4 ml in the area of middle third of the neck subcutaneously in line with the experimental design presented on Figure 1. Three weeks later, blood samples were collected to obtain serum and analyze rabies virus-neutralizing antibodies presence using the FAVN test (Astray et al., 2017).

**Figure 4:**
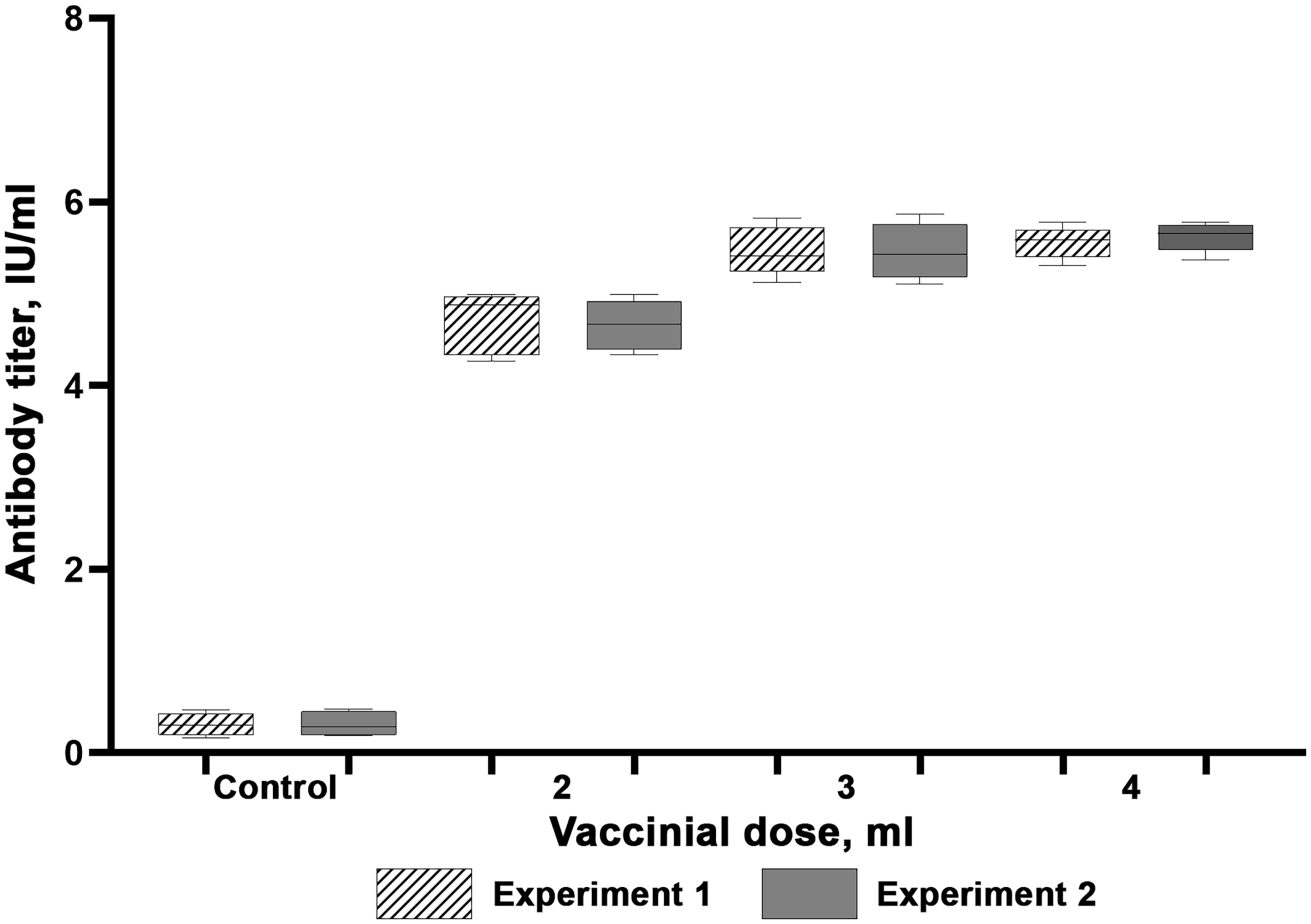
Virus-neutralizing activity of blood serum of reindeer once immunized with the Liquid vaccine. Two independent experiments (Experiment 1 and Experiment 2). In each experiment, 15 animals were vaccinated (five animals per each dose). Four unvaccinated animals were used as a control group. Reindeer blood serum were collected 21 days after administration. Maximum and minimum titres are illustrated with whisker caps, interquartile range with a box with the median denoted by a horizontal line.

**Figure 5:**
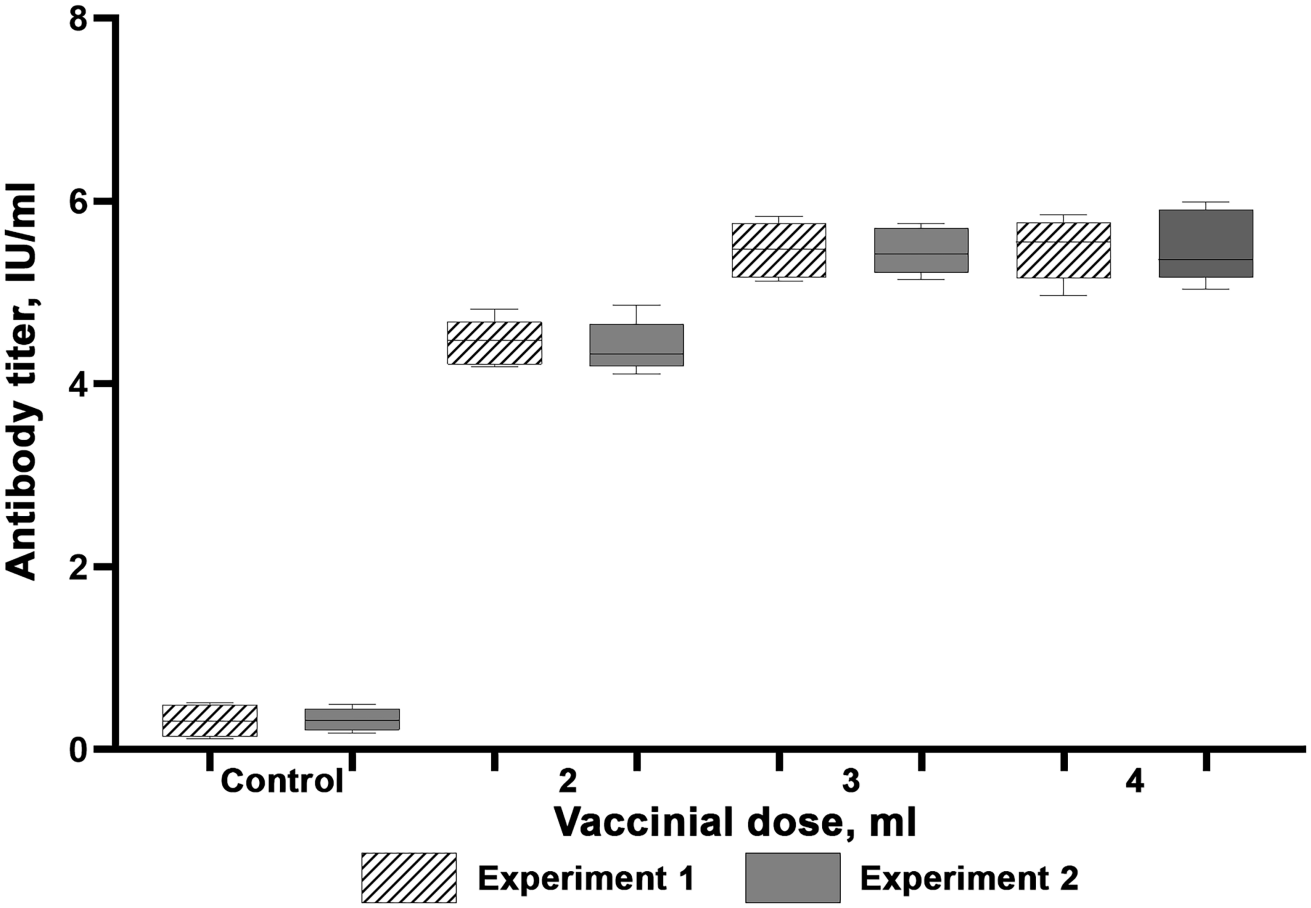
Virus-neutralizing activity of blood serum of reindeer once immunized with the Lyophilized vaccine. Two independent experiments (Experiment 1 and Experiment 2). In each experiment, 15 animals were vaccinated (5 animals per each dose). Four unvaccinated animals were used as a control group. Reindeer blood serum were collected 21 days after administration. Maximum and minimum titres are illustrated with whisker caps, interquartile range with a box with the median denoted by a horizontal line.

The results of experiments to determine rabies vaccine immunizing doses are available as Figures 4, 5. Our studies led to a conclusion that both rabies vaccines induced protective immunity in reindeer (antibody titers higher than 0.5 IU/ml) at day 21 post-vaccination, which confirms their compliance with OIE antigenic activity requirements. Four unvaccinated animals kept their antibody titers on the initial level below 0.25 IU/ml. These results suggest that the dose of 3 ml is optimal for immunizing with the Liquid Vaccine since the 4 ml dose does not considerably amplify antibody titers as compared to the 3 ml dose. In the same manner as for the Liquid Vaccine, the results of the study suggest that the dose of 3 ml is optimal when immunizing with the Lyophilized Vaccine since the 4 ml dose does not considerably amplify antibody titers as compared to the 3 ml dose.

### Reindeer immunity level evaluation after double vaccination against rabies

Forty animals were used to evaluate the immunity level after single and double vaccination with the Liquid and Lyophilized Vaccines in doses of 3 ml administered subcutaneously. The experimental design is available as Figure 2. The second vaccination was made on 30 day after the first one.

**Figure 6:**
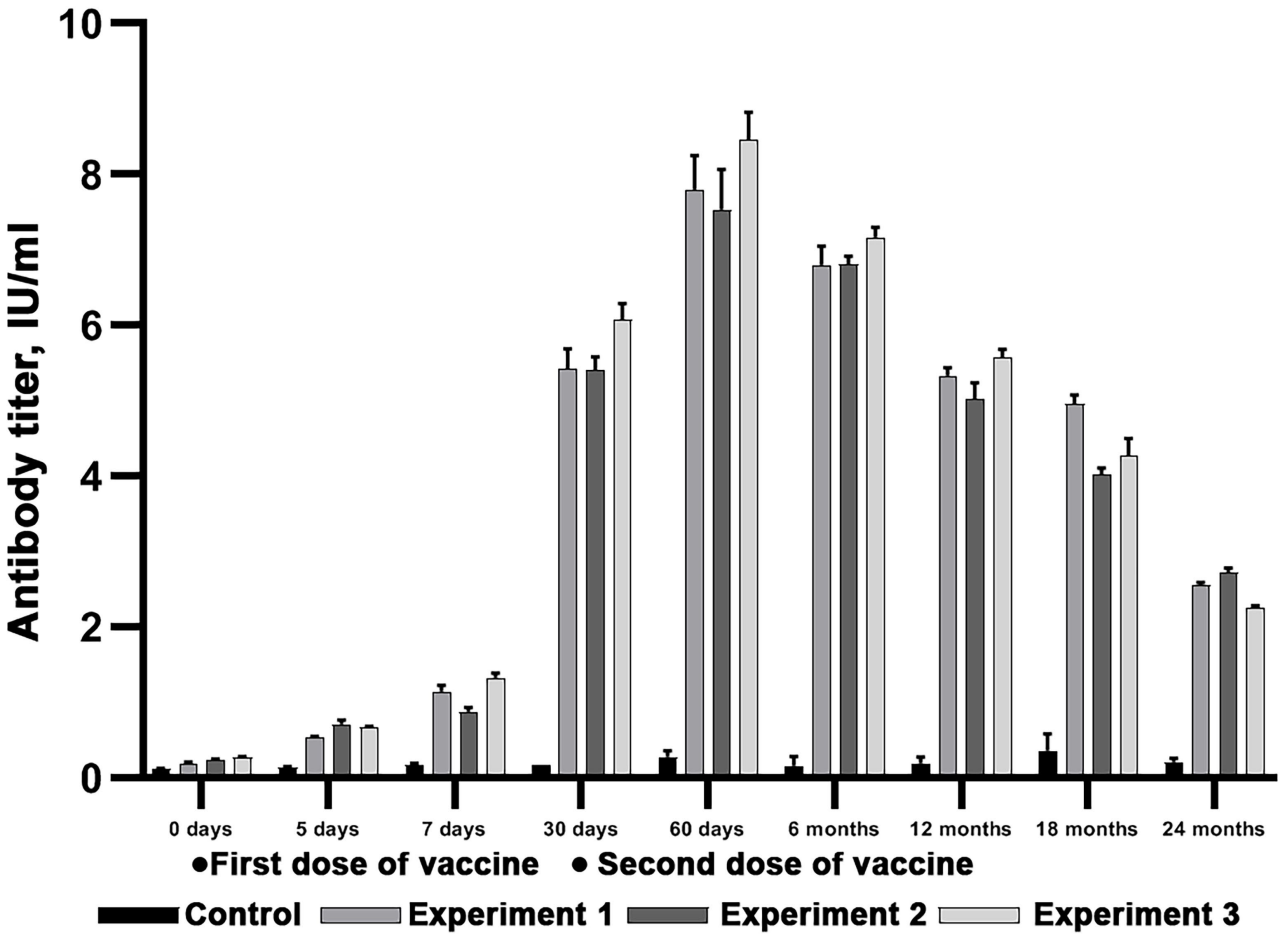
Evaluation of virus-neutralizing antibody titers in reindeer blood serum after double immunization with Lyophilized vaccine. Three independent experiments (Experiment 1, Experiment 2 and Experiment 3). In each experiment, six animals were vaccinated in doses equal to 3 ml. Two unvaccinated animals were used as a control group. The standard deviation is illustrated with whisker caps.

**Figure 7:**
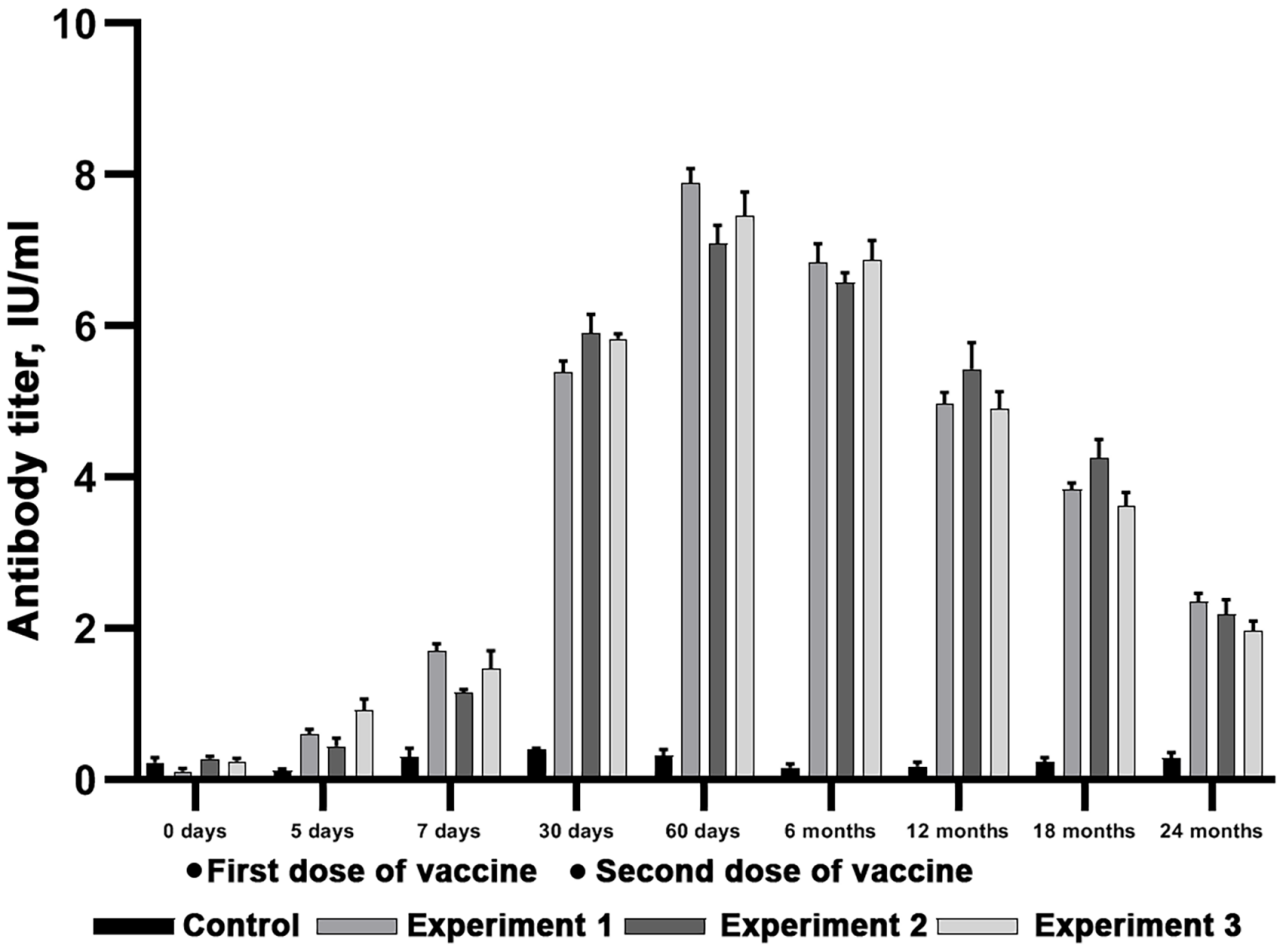
Evaluation of virus-neutralizing antibody titers in reindeer blood serum after double immunization with Liquid vaccine. Three independent experiments (Experiment 1, Experiment 2 and Experiment 3). In each experiment, 6 animals were vaccinated in doses equal to 3 ml. Two unvaccinated animals were used as a control group. The standard deviation is illustrated with whisker caps.

The results presented on Figures 6, 7 suggest that both vaccines induce a significant protective virus-neutralizing antibody titers. At day 7 and after 24 months after the start of the study the antibody level in all experimental groups surpassed the threshold of 0.5 IU/ml (Wilcoxon signed-rank test, value of *p* < 0.05), which fits OIE requirements. The tables of sera titers from each reindeer are presented in Supplementary material (Supplementary Tables 1–4).

### Effectiveness of reindeer vaccination against rabies in Russia (field trials)

Rabies vaccines were applied at Taba-Yana reindeer farms in the Ust-Yansk District of the Republic of Sakha (Yakutia) from 2012 to 2017 to protect animals against rabies in field conditions. Reindeer were fed by the open grazing system. Over that five-year observation period, 4,359 animals were double vaccinated (30 days interval), and 1,953 blood serum samples were collected 30 days post vaccination. The samples were analyzed to evaluate virus-neutralizing antibody titers using the FAVN test.

For the Liquid Vaccine, antigenic activity assessment in field conditions showed that the seroconversion rate of reindeer blood serum amounted to about 92.3% with the mean antibody titer of 6.58 ± 1.36 IU/ml (1,053 samples were tested). As many as 90.9% of the animals immunized with the Lyophilized Vaccine showed virus-neutralizing antibody levels that are appropriate for protection from the disease. The mean antibody titer summed up to 6.08 ± 1.43 IU/ml (Table 3).

**Table 3:** Immune status of reindeer vaccinated against rabies.

Vaccine	Number of animals	Mean antibody titer (IU/ml)^*^	Protected animals (%)
Liquid vaccine	1,053	6.58 ± 1.36	92.3
Lyophilized vaccine	900	6.08 ± 1.43	90.9
Total/mean	1,953	6.33 ± 1.395	91.6

* According to international standards, an animal is protected from rabies if the rabies antibody level in its blood equals or exceeds 0.5 IU/ml.

**Figure 8:**
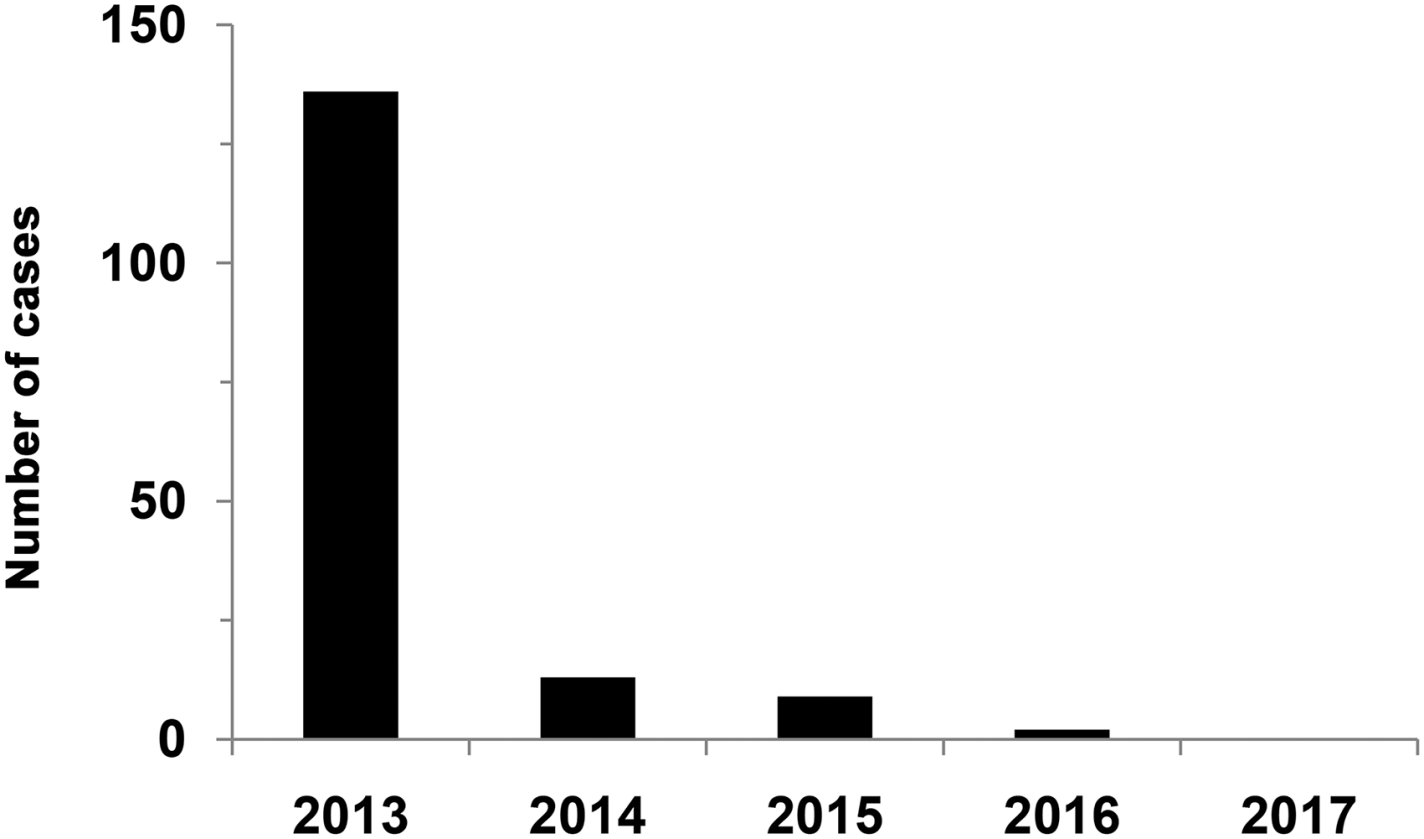
Rabies incidence in reindeer in post-vaccination period. The data were taken from the official annual report on the epizootological situation with extra dangerous diseases in Russia in 2013–2017.

Indirect evidence of the effectiveness of vaccines are data of the incidence of rabies during the program for vaccination of deer. Figure 8 illustrates the effectiveness of reindeer vaccination against rabies. It is established that the number of diseased animals fell down to zero in that period thanks to preventive actions and vaccination campaigns.

## Discussion

Reindeer herding is a traditional occupation of the indigenous peoples of the North, Siberia and the Far East, an integral part of their national culture and way of surviving in harsh climatic conditions. There are over 1.7 million domestic deer or about 2/3 of the world’s domestic reindeer in the Russian Federation (Ivanov and Shcherbakova, 2021).

The rabies-related epizootological situation in the Russian Federation has become tense in the past decade, as evidenced by detected rabies cases among reindeer populations. Rabies foci are distributed non-homogeneously across Russian regions, with this distribution varies significantly year to year (Layshev et al., 2015). In particular, reindeer rabies cases have been observed in the Republics of Komi and Sakha (Yakutia), in the Nenets and Yamalo-Nenets Autonomous Districts, on Taimyr and Chukotka. Most frequently, rabies strikes foxes, polar foxes, wolves and wolverines. The Animal Health Office detects the disease toward the end of winter and early spring (February–March) while animals migrate and copulate actively. Cases of the disease among domestic deer indicate the transmission of the rabies by representatives of the wild fauna through reindeer dogs, after contact with infected arctic foxes that accompany herds of domestic reindeer in the winter–spring period. Also, wolverines with rabies often attack deer. As reindeer are pastured in open terrain, where there is a higher chance of wild predator offence and consequently a higher chance of infection with rabies virus, the strategy of combating reindeer rabies has to rely heavily on regular administration of an injection vaccine to achieve and maintain a vaccination coverage required to cease rabies virus transmission. The use of baits for oral immunization by live vaccines leads to the elimination of rabies from the population of wild animals. For example, the use of safe and potent rabies vaccines such as live attenuated rabies virus vaccine SAG2 based on SAD Bern virus strain largely contributed to the elimination of rabies in several European countries (Mähl et al., 2014). Since the end of the 20th century, foreign bait vaccines based on viral strains SAD Bern and SAD B19 have been used in Russia for oral immunization of wild animals (Rupprecht and Plotkin, 2012). Later, Russian oral vaccines developed using attenuated rabies virus strains RB-71, TS-80, Vnukovo-32/07, RV-97, and ERA-G333 were used (Elakov, 2022). Viral vector vaccines based on poxvirus, adenovirus, and parainfluenza expressing the G protein of the rabies virus have been successfully used to prevent rabies at present (Yarosh et al., 1996; Chen et al., 2013; Stading et al., 2017). For example, the RABORAL V-RG rabies vaccine based on a vaccinia virus vector is used for oral vaccination of red foxes, golden jackals and raccoon dogs (Cliquet et al., 2008). Viral vector rabies vaccines based on various strains of adenovirus are also currently used to vaccinate animals (Fehlner-Gardiner et al., 2012; Shen et al., 2012; Yang et al., 2013).

The air temperature in the Republic of Sakha (Yakutia) varies from +20 °С to −35 °С over the course of the year, therefore it is difficult to meet temperature requirements for storage and application for the Liquid Vaccine. That is the reason why the Lyophilized vaccine is also used, as it can be stored and transported at any freezing temperatures. Furthermore, unlike the Lyophilized Vaccine, the Liquid Vaccine additionally contains aluminum hydroxide as an adjuvant. This is the rationale behind our decision to study effectiveness of both rabies vaccines based on virus-neutralizing antibody titers that were determined using the internationally accepted method of FAVN. This method measures the immune system response to rabies vaccine administration and allows for analyzing blood serum antibody levels. This type of neutralization reaction is widely used to examine blood serum samples collected from dogs and cats that are to be transported abroad, and to perform international comparison tests.

Since the first rabies vaccine development by Louis Pasteur in the late 19^th^ century vaccination has been used widely to immunize domestic animals. Current vaccine manufacturers employ a variety of fixed vaccine rabies virus strains, namely Pasteur virus (PV), Pitman Moore (PM), Challenge Virus Standard (CVS), Flury low egg passage (LEP), Flury high egg passage (HEP), Kelev, Evelyn Rokitniki Abelseth (ERA), and Street Alabama Dufferin (SAD). These strains are used many countries, and new strain derivatives are obtained in the course of adaptation to manufacturing conditions. Table 4 represents a summary of well-known rabies vaccine characteristics.

**Table 4:** Summary of characteristics of current rabies vaccines.

Manufacturer	Zoetis (United States)	Intervet (Netherlands)	Virbac SA (France)	Boehringer Ingelheim (France)
Commercial name	Defensor^®^3	Nobivac^®^ Rabies	Rabigen^®^ Mono	Rabisin^®^
International nonproprietary name (INN)	Inactivated rabies vaccine for dogs, cats, cattle and sheep	Inactivated animal rabies vaccine	Vaccine for active immunization of dogs and cats against rabies	Inactivated rabies vaccine for the immunization of dogs, cats, horses, cattle, sheep and goats
Strain	PV: Paris	Pasteur RIV	VR-12	G-52
Pharmaceutical form	Suspension	Suspension	Suspension	Suspension
Animal species	Cattle, sheep, dogs, cats, polecats	Cattle, horses, dogs, cats	Dogs, cats	Cattle, horses, sheep, dogs, cats, polecats
Immunogenic activity	Not specified	No less than 2 IU	No less than 1 IU	No less than 1 IU

**Figure 9:**
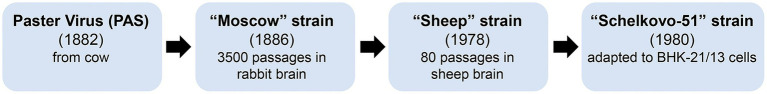
Generation of manufacturing rabies virus strains used in Russian rabies vaccines.

In this work, inactivated vaccines based on the highly infectious strain Schelkovo-51 were used. A brief diagram of Shchelkovo-51 generation is available as Figure 9. The Louis Pasteur virus (PAS) was isolated from a cow by Louis Pasteur in France in 1882. This strain was passaged 2,061 times in rabbits, adapted to the Vero cell culture (19 passages), then to the BSR cell culture (5 passages), and then it was used to produce veterinary and medical rabies vaccines. The strain was delivered to Russia in 1886 where it became a progenitor of several vaccine strains. The Moscow strain was obtained after virus attenuation in rabbits for 3,500 passages. Passaging led to acquiring the Sheep strain from the Moscow strain; the former is an ancestor of two manufacturing rabies virus strains widely known in Russia: Shchelkovo-51 and PV-97. The Shchelkovo-51 vaccine rabies virus strain was adapted to the BHK-21 suspension cell line from golden hamster kidney, and is highly immunogenic.

Rabies glycoprotein G is the only viral protein exposed on the virus surface. Previous studies showed that glycoprotein G is a protective antigen that is able to induce synthesis of virus-neutralizing antibodies (VNA), which are responsible for immune reactions of the host (Astray et al., 2017). The GenBank database contains no data on full-genome sequencing of the Shchelkovo-51 vaccine strain. Given the special importance of rabies virus glycoprotein G as a protective antigen, we decided to sequence this exact genome fragment to evaluate the level of homology of its nucleotide sequences and those of other known rabies virus strains. An analysis of nucleotide sequences of the Shchelkovo-51 rabies virus strain showed that their homology with sequences of other rabies virus strains ranged from 93 to 99%. It is worth noting that glycoprotein G nucleotide sequences homology of the Shchelkovo-51 virus strain and the challenge virus standard (CVS-11) strain used as a laboratory standard in rabies reference centers was 89%. We conducted the study of genetic stability of the Shchelkovo-51 rabies virus strain at different stages of vaccine manufacturing by comparing glycoprotein G sequences of the strain at passages 2 and 16. It was established that the glycoprotein G-coding RNA sequence of the Shchelkovo-51 strain remained 100% identical at these passages. This data makes it possible to confirm a high level of stability of the strain at manufacturing process stages, at least as far as the fragment under consideration is concerned.

Two inactivated commercial vaccines based on the Shchelkovo-51 rabies virus strain widely used in Russia for the vaccination of pets and bovine animals were used in this study to achieve the protective level of humoral immunity against rabies (≥0.5 IU/сm^3^) in reindeer. Both vaccines have high safety and efficiency levels for the target animal species. These vaccines promoted development of protective virus-neutralizing antibody titers being administered in the dose equal to 3.0 ml, and satisfied OIE requirements. A single dose of the vaccines induced stable production of specific neutralizing antibodies in reindeer already at day-7 post-vaccination, and at day-30 the antibody titers significantly surpassed the protective threshold determined by the OIE. Double administration kept the protective level of antibodies for 24 months. These results are in line with the earlier data of Sihvonen et al. (1993) demonstrated induction of good but short-duration immunity in reindeer vaccinated by one dose of inactivated adjuvanted rabies vaccine. According to their data, antibody titres were not found 1 year after primary vaccination in most animals and a booster dose of the vaccine is needed to provide adequate levels of protection. Our results indicate that a booster dose of the vaccine is needed 30 days after the primary vaccination to ensure sufficient protection for at least 2 years.

Vaccine effectiveness in reindeer was a subject of years-long research. In field trials the presented vaccines were proven to be harmless and able to induce a strong immunity in vaccinated animals. Confirmation of the protective effect of vaccines is also the reduction in the incidence of rabies among reindeer during the testing period.

Surprisingly, the effectiveness of adjuvanted Liquid vaccine and non-adjuvanted Lyophilized vaccine was close. Thus, aluminum hydroxide did not stimulate the production of a higher titer of virus-neutralizing antibodies. Aluminum hydroxide is one of the classic adjuvants and is found in many protein subunit and inactivated vaccines. However, in recent years, data have been accumulating on the poor immunostimulatory activity of aluminum salts (McKee et al., 2009; D’Souza et al., 2013; Trifonova et al., 2017; Kurashova et al., 2020). Our results are in line with this data and do not support the benefit of aluminum hydroxide as an adjuvant in inactivated vaccine for the prevention of rabies in reindeer.

Based on results of this study, the following data were added to the vaccine administration manual: both vaccines are recommended for subcutaneous double immunization (at 30 days interval) of reindeer in the dose of 3.0 ml, which provides for efficient prevention of this dangerous disease in the Arctic. Our study shows that both Liquid and Lyophilized Vaccines are safe for reindeer and can be recommended for use.

Effective vaccination programs are essential to eliminate rabies from wildlife in a limited area. In this way, successful experience with rabies vaccines based on the Schelkovo-51 virus strain for the protection of reindeer should be complemented by the use of oral rabies vaccine baits based on attenuated live virus or virus vector in other animals such as red foxes, arctic foxes and wolverines.

## Data availability statement

We confirm that our data (GenBank Acces. num. ON181556) is associated with this publication.

## Author contributions

YF, OA, AZ, and VY designed the research. IL, NM, RM, EM, and KA carried out the experiments. OK, NN, and IM contributed to data analysis and wrote the manuscript. All authors contributed to the article and approved the submitted version.

## Funding

This work was partially funded by grant no. 075–15–2021-1054 given by the Russian Science and Higher Education Ministry to facilitate specific actions in the framework of the 2019–2027 Federal Scientific and Technical Program for Genetic Technologies Development.

## Conflict of interest

The authors declare that the research was conducted in the absence of any commercial or financial relationships that could be construed as a potential conflict of interest.

## Publisher’s note

All claims expressed in this article are solely those of the authors and do not necessarily represent those of their affiliated organizations, or those of the publisher, the editors and the reviewers. Any product that may be evaluated in this article, or claim that may be made by its manufacturer, is not guaranteed or endorsed by the publisher.
