# Supplementary material for: Long-term humoral immunogenicity, safety and protective efficacy of inactivated vaccine against reindeer rabies
Source: Front Microbiol. 2022 Sep 8;13:988738. doi: 10.3389/fmicb.2022.988738 (PMC9493026; doi:10.3389/fmicb.2022.988738)
Supplement: Supplementary file 1 [file Table_1.DOCX]

|  | **Dose. ml** | **Animal number** | | | | |
| --- | --- | --- | --- | --- | --- | --- |
| **Experiment 1** |  | 1 | 2 | 3 | 4 | 5 |
|  | 2 | 4.27 | 4.95 | 4.39 | 4.88 | 4.99 |
|  | 3 | 5.12 | 5.62 | 5.36 | 5.82 | 5.41 |
|  | 4 | 5.62 | 5.49 | 5.78 | 5.31 | 5.59 |
|  | Control | 0.16 | 0.31 | 0.47 | 0.29 |  |
|  |  |  |  |  |  |  |
| **Experiment 2** |  | 6 | 7 | 8 | 9 | 10 |
|  | 2 | 4.34 | 4.85 | 4.45 | 4.67 | 4.99 |
|  | 3 | 5.43 | 5.87 | 5.25 | 5.64 | 5.11 |
|  | 4 | 5.37 | 5.58 | 5.78 | 5.71 | 5.66 |
|  | Control | 0.20 | 0.37 | 0.19 | 0.48 |  |

**Supplementary Table 1**. Virus-neutralizing activity of blood serum of reindeer once immunized with the Liquid vaccine. Two independent experiments (Experiment 1 and Experiment 2).
