# Supplementary material for: Long-term humoral immunogenicity, safety and protective efficacy of inactivated vaccine against reindeer rabies
Source: Front Microbiol. 2022 Sep 8;13:988738. doi: 10.3389/fmicb.2022.988738 (PMC9493026; doi:10.3389/fmicb.2022.988738)
Supplement: Supplementary file 2 [file Table_2.DOCX]

|  | **Dose. ml** | **Animal number** | | | | |
| --- | --- | --- | --- | --- | --- | --- |
| **Experiment 1** |  | 1 | 2 | 3 | 4 | 5 |
|  | 2 | 4.48 | 4.23 | 4.54 | 4.82 | 4.19 |
|  | 3 | 5.12 | 5.69 | 5.47 | 5.21 | 5.83 |
|  | 4 | 5.33 | 5.85 | 4.97 | 5.68 | 5.55 |
|  | Control | 0.12 | 0.43 | 0.19 | 0.51 |  |
|  |  |  |  |  |  |  |
| **Experiment 2** |  | 6 | 7 | 8 | 9 | 10 |
|  | 2 | 4.86 | 4.33 | 4.11 | 4.46 | 4.28 |
|  | 3 | 5.42 | 5.14 | 5.66 | 5.75 | 5.29 |
|  | 4 | 5.36 | 5.83 | 5.99 | 5.29 | 5.04 |
|  | Control | 0.18 | 0.33 | 0.49 | 0.31 |  |

**Supplementary Table 2**. Virus-neutralizing activity of blood serum of reindeer once immunized with the Lyophilized vaccine. Two independent experiments (Experiment 1 and Experiment 2).
