# Supplementary material for: Long-term humoral immunogenicity, safety and protective efficacy of inactivated vaccine against reindeer rabies
Source: Front Microbiol. 2022 Sep 8;13:988738. doi: 10.3389/fmicb.2022.988738 (PMC9493026; doi:10.3389/fmicb.2022.988738)
Supplement: Supplementary file 3 [file Table_3.DOCX]

|  | 0 days | 5 days | 7 days | 30 days | 60 days | 6 month | 12 month | 18 month | 24 month |
| --- | --- | --- | --- | --- | --- | --- | --- | --- | --- |
| **Experiment 1** | 0.18 | 0.53 | 1.06 | 5.38 | 7.84 | 6.68 | 5.33 | 4.85 | 2.59 |
|  | 0.21 | 0.52 | 1.05 | 5.54 | 7.17 | 6.85 | 5.4 | 5.07 | 2.57 |
|  | 0.17 | 0.51 | 1.09 | 5.22 | 8.49 | 7.24 | 5.16 | 4.88 | 2.56 |
|  | 0.18 | 0.53 | 1.14 | 5.88 | 7.68 | 6.83 | 5.48 | 5.13 | 2.52 |
|  | 0.17 | 0.56 | 1.22 | 5.36 | 7.54 | 6.49 | 5.27 | 4.85 | 2.52 |
|  | 0.21 | 0.53 | 1.26 | 5.16 | 8.05 | 6.66 | 5.33 | 4.93 | 2.58 |
|  | | | | | | | | | |
| **Experiment 2** | 0.21 | 0.72 | 0.81 | 5.44 | 8.01 | 6.87 | 4.71 | 4.09 | 2.83 |
|  | 0.24 | 0.60 | 0.83 | 5.57 | 6.63 | 6.77 | 4.86 | 3.98 | 2.71 |
|  | 0.20 | 0.76 | 0.97 | 5.26 | 7.89 | 6.85 | 5.09 | 4.14 | 2.68 |
|  | 0.22 | 0.69 | 0.88 | 5.16 | 7.20 | 6.91 | 5.08 | 4.02 | 2.67 |
|  | 0.24 | 0.72 | 0.85 | 5.40 | 7.56 | 6.82 | 5.32 | 3.91 | 2.62 |
|  | 0.24 | 0.74 | 0.89 | 5.60 | 7.90 | 6.61 | 5.08 | 3.97 | 2.74 |
|  | | | | | | | | | |
| **Experiment 3** | 0.26 | 0.68 | 1.35 | 5.70 | 8.93 | 7.01 | 5.45 | 3.98 | 2.22 |
|  | 0.28 | 0.66 | 1.37 | 6.28 | 7.95 | 7.12 | 5.65 | 4.66 | 2.20 |
|  | 0.26 | 0.68 | 1.37 | 6.05 | 8.45 | 7.05 | 5.49 | 4.35 | 2.23 |
|  | 0.28 | 0.67 | 1.35 | 6.27 | 8.20 | 7.22 | 5.71 | 4.29 | 2.29 |
|  | 0.25 | 0.65 | 1.21 | 6.13 | 8.81 | 7.24 | 5.47 | 4.14 | 2.28 |
|  | 0.24 | 0.67 | 1.29 | 5.99 | 8.37 | 7.35 | 5.63 | 4.17 | 2.25 |
|  | | | | | | | | | |
| **C*** | 0.11 | 0.14 | 0.18 | 0.16 | 0.33 | 0.24 | 0.13 | 0.19 | 0.24 |
|  | 0.12 | 0.11 | 0.14 | 0.16 | 0.21 | 0.07 | 0.25 | 0.51 | 0.16 |

***** control unvaccinated group

**Supplementary Table 3**. Evaluation of virus-neutralizing antibody titers in reindeer blood serum after double immunization with Lyophilized vaccine. Three independent experiments (Experiment 1, Experiment 2 and Experiment 3).
