# Supplementary material for: Long-term humoral immunogenicity, safety and protective efficacy of inactivated vaccine against reindeer rabies
Source: Front Microbiol. 2022 Sep 8;13:988738. doi: 10.3389/fmicb.2022.988738 (PMC9493026; doi:10.3389/fmicb.2022.988738)
Supplement: Supplementary file 4 [file Table_4.DOCX]

|  | 0 days | 5 days | 7 days | 30 days | 60 days | 6 month | 12 month | 18 month | 24 month |
| --- | --- | --- | --- | --- | --- | --- | --- | --- | --- |
| **Experiment 1** | 0.05 | 0.52 | 1.56 | 5.29 | 7.99 | 6.43 | 4.91 | 3.92 | 2.41 |
|  | 0.17 | 0.67 | 1.67 | 5.55 | 8.06 | 6.92 | 4.70 | 3.79 | 2.41 |
|  | 0.08 | 0.61 | 1.79 | 5.48 | 7.86 | 7.10 | 4.96 | 3.86 | 2.45 |
|  | 0.11 | 0.68 | 1.57 | 5.33 | 8.09 | 6.68 | 5.18 | 3.81 | 2.24 |
|  | 0.12 | 0.54 | 1.79 | 5.15 | 7.70 | 7.04 | 5.08 | 3.94 | 2.39 |
|  | 0.07 | 0.57 | 1.77 | 5.50 | 7.63 | 6.85 | 4.92 | 3.72 | 2.16 |
|  | | | | | | | | | |
| **Experiment 2** | 0.26 | 0.27 | 1.13 | 5.55 | 7.01 | 6.43 | 5.03 | 3.98 | 2.10 |
|  | 0.25 | 0.40 | 1.21 | 6.04 | 6.63 | 6.68 | 5.82 | 4.31 | 2.12 |
|  | 0.33 | 0.48 | 1.18 | 5.86 | 7.09 | 6.52 | 5.10 | 4.64 | 2.20 |
|  | 0.27 | 0.40 | 1.08 | 6.02 | 7.20 | 6.43 | 5.53 | 4.28 | 2.04 |
|  | 0.28 | 0.50 | 1.12 | 5.73 | 7.26 | 6.58 | 5.83 | 4.30 | 2.57 |
|  | 0.22 | 0.58 | 1.15 | 6.24 | 7.31 | 6.76 | 5.28 | 3.99 | 2.05 |
|  | | | | | | | | | |
| **Experiment 3** | 0.20 | 1.01 | 1.35 | 5.71 | 7.15 | 6.65 | 4.51 | 3.50 | 1.81 |
|  | 0.22 | 0.91 | 1.87 | 5.82 | 7.56 | 7.16 | 4.86 | 3.59 | 2.03 |
|  | 0.26 | 1.15 | 1.57 | 5.82 | 8.03 | 6.66 | 4.94 | 3.67 | 2.04 |
|  | 0.29 | 0.71 | 1.35 | 5.87 | 7.45 | 6.72 | 5.10 | 3.43 | 1.92 |
|  | 0.26 | 0.85 | 1.21 | 5.90 | 7.30 | 7.22 | 4.78 | 3.95 | 1.90 |
|  | 0.21 | 0.83 | 1.49 | 5.86 | 7.23 | 6.83 | 5.16 | 3.55 | 2.14 |
| **C*** | 0.27 | 0.13 | 0.38 | 0.41 | 0.37 | 0.11 | 0.13 | 0.27 | 0.22 |
|  | 0.17 | 0.10 | 0.21 | 0.39 | 0.26 | 0.19 | 0.21 | 0.18 | 0.33 |

***** control unvaccinated group

**Supplementary Table 4**. Evaluation of virus-neutralizing antibody titers in reindeer blood serum after double immunization with Lyophilized vaccine. Three independent experiments (Experiment 1, Experiment 2 and Experiment 3).
