# Supplementary material for: Long-term humoral immunogenicity, safety and protective efficacy of inactivated vaccine against reindeer rabies
Source: Front Microbiol. 2022 Sep 8;13:988738. doi: 10.3389/fmicb.2022.988738 (PMC9493026; doi:10.3389/fmicb.2022.988738)
Supplement: Supplementary file 5 [file Data_Sheet_1.DOCX]

2 pass. 1 TTATGGTCTGACATGTCTCTTCAGACACAAAGGTCTGAAGAGGACAAAGATTCCTCTCTG 60

||||||||||||||||||||||||||||||||||||||||||||||||||||||||||||

16 pass. 1 TTATGGTCTGACATGTCTCTTCAGACACAAAGGTCTGAAGAGGACAAAGATTCCTCTCTG 60

2 pass. 61 CTTCTAGAATAATCAGATCATGTCCCCAAAGTTCATCACTTGCATACCTCTGGAGAAGAG 120

||||||||||||||||||||||||||||||||||||||||||||||||||||||||||||

16 pass. 61 CTTCTAGAATAATCAGATCATGTCCCCAAAGTTCATCACTTGCATACCTCTGGAGAAGAG 120

2 pass. 121 AACACACGGGCTTAACTTCAACCCTTGGGAGCAATAGAACAAAAACATGTTATGGTACCA 180

||||||||||||||||||||||||||||||||||||||||||||||||||||||||||||

16 pass. 121 AACACACGGGCTTAACTTCAACCCTTGGGAGCAATAGAACAAAAACATGTTATGGTACCA 180

2 pass. 181 TTCAACCGCTGCATTTTATCAGAGTTAAGTTGATTGCCTTTACATTTTGAGCCTCCTAGA 240

||||||||||||||||||||||||||||||||||||||||||||||||||||||||||||

16 pass. 181 TTCAACCGCTGCATTTTATCAGAGTTAAGTTGATTGCCTTTACATTTTGAGCCTCCTAGA 240

2 pass. 241 TGCGAAAAAAACTATTAACATCCCTCAAAAGACTTAGGGAAAAATGGTTCCTCAGGCTCT 300

||||||||||||||||||||||||||||||||||||||||||||||||||||||||||||

16 pass. 241 TGCGAAAAAAACTATTAACATCCCTCAAAAGACTTAGGGAAAAATGGTTCCTCAGGCTCT 300

2 pass. 301 TTTGTTTGTACCCTTTCTGGGTTTTTCATTGTGTTTCGGGAAATTCCCTATTTACACGAT 360

||||||||||||||||||||||||||||||||||||||||||||||||||||||||||||

16 pass. 301 TTTGTTTGTACCCTTTCTGGGTTTTTCATTGTGTTTCGGGAAATTCCCTATTTACACGAT 360

2 pass. 361 ACCGGACAAACTTGGTCCCTGGAGCCCGATTGATATACATCATCTCAGTTGCCCAAACAA 420

||||||||||||||||||||||||||||||||||||||||||||||||||||||||||||

16 pass. 361 ACCGGACAAACTTGGTCCCTGGAGCCCGATTGATATACATCATCTCAGTTGCCCAAACAA 420

2 pass. 421 TTTGGTCGTGGAGGATGAAGGATGCACCAACCTGTCAGGGTTCTCCTACATGGAACTTAA 480

||||||||||||||||||||||||||||||||||||||||||||||||||||||||||||

16 pass. 421 TTTGGTCGTGGAGGATGAAGGATGCACCAACCTGTCAGGGTTCTCCTACATGGAACTTAA 480

2 pass. 481 AGTTGGATACATCTCTGCTATAAAGGTGAACGGATTCACTTGCACAGGCGTTGTGACAGA 540

||||||||||||||||||||||||||||||||||||||||||||||||||||||||||||

16 pass. 481 AGTTGGATACATCTCTGCTATAAAGGTGAACGGATTCACTTGCACAGGCGTTGTGACAGA 540

2 pass. 541 GGCAGAGACCTACACTAACTTTGTTGGTTATGTCACCACCACGTTCAAAAGAAAGCATTT 600

||||||||||||||||||||||||||||||||||||||||||||||||||||||||||||

16 pass. 541 GGCAGAGACCTACACTAACTTTGTTGGTTATGTCACCACCACGTTCAAAAGAAAGCATTT 600

2 pass. 601 CCGCCCGACACCAGATGCATGTAGAGCCGCATACAACTGGAAGACGGCTGGTGATCCCAG 660

||||||||||||||||||||||||||||||||||||||||||||||||||||||||||||

16 pass. 601 CCGCCCGACACCAGATGCATGTAGAGCCGCATACAACTGGAAGACGGCTGGTGATCCCAG 660

2 pass. 661 ATATGAAGAGTCTTTACAMAATCCGTACCCTGACTACCAGTGGCTCCGAACTGTAAGAAC 720

||||||||||||||||||||||||||||||||||||||||||||||||||||||||||||

16 pass. 661 ATATGAAGAGTCTTTACAMAATCCGTACCCTGACTACCAGTGGCTCCGAACTGTAAGAAC 720

2 pass. 721 CACCAAGGAGTCTCTCGTTATCATATCCCCAAGTGCGGCAGATTTGGACCCATATGACAA 780

||||||||||||||||||||||||||||||||||||||||||||||||||||||||||||

16 pass. 721 CACCAAGGAGTCTCTCGTTATCATATCCCCAAGTGCGGCAGATTTGGACCCATATGACAA 780

2 pass. 781 ATCCCTTCACTCGAGGGTCTTCCCTAGCGGAAAGTGCTCAGGAATAACGGTGTCCTCTGT 840

||||||||||||||||||||||||||||||||||||||||||||||||||||||||||||

16 pass. 781 ATCCCTTCACTCGAGGGTCTTCCCTAGCGGAAAGTGCTCAGGAATAACGGTGTCCTCTGT 840

2 pass. 841 TTACTGCTCAACAAACCACGATTACACCATTTGGATGCCTGAGAATCCGAGACAAGGGAT 900

||||||||||||||||||||||||||||||||||||||||||||||||||||||||||||

16 pass. 841 TTACTGCTCAACAAACCACGATTACACCATTTGGATGCCTGAGAATCCGAGACAAGGGAT 900

2 pass. 901 GTCTTGTGACATTTTCACCAATAGTAGAGGGAAGAGAGCATCCAAGGAGAGCAAGACCTG 960

||||||||||||||||||||||||||||||||||||||||||||||||||||||||||||

16 pass. 901 GTCTTGTGACATTTTCACCAATAGTAGAGGGAAGAGAGCATCCAAGGAGAGCAAGACCTG 960

2 pass. 961 CGGCTTTGTGGATGAAAGAGGCCTGTATAAGTCTCTAAGAGGCTCATGCAAACTCAAGTT 1020

||||||||||||||||||||||||||||||||||||||||||||||||||||||||||||

16 pass. 961 CGGCTTTGTGGATGAAAGAGGCCTGTATAAGTCTCTAAGAGGCTCATGCAAACTCAAGTT 1020

2 pass. 1021 ATGTGGAGTTCTTGGACTTAGACTTATGGATGGAACATGGGTCGCGATGCAGACATCAAA 1080

||||||||||||||||||||||||||||||||||||||||||||||||||||||||||||

16 pass. 1021 ATGTGGAGTTCTTGGACTTAGACTTATGGATGGAACATGGGTCGCGATGCAGACATCAAA 1080

2 pass. 1081 TGAGACCAAATGGTGTTCCCCTGATCAGTTGGTTAATCTGCACGACTTTCACTCAGATGA 1140

||||||||||||||||||||||||||||||||||||||||||||||||||||||||||||

16 pass. 1081 TGAGACCAAATGGTGTTCCCCTGATCAGTTGGTTAATCTGCACGACTTTCACTCAGATGA 1140

2 pass. 1141 AATTGAGCATCTTGTTGTAGAGGAGTTGGTCAAGAAAAGAGAGGAGTGTCTGGATGCACT 1200

||||||||||||||||||||||||||||||||||||||||||||||||||||||||||||

16 pass. 1141 AATTGAGCATCTTGTTGTAGAGGAGTTGGTCAAGAAAAGAGAGGAGTGTCTGGATGCACT 1200

2 pass. 1201 AGAGTCCATCATGACCACCAAGTCAGTAAGTTTCAGACGTCTCAGTCATTTAAGAAAACT 1260

||||||||||||||||||||||||||||||||||||||||||||||||||||||||||||

16 pass. 1201 AGAGTCCATCATGACCACCAAGTCAGTAAGTTTCAGACGTCTCAGTCATTTAAGAAAACT 1260

2 pass. 1261 TGTCCCTGGGTTCGGAAAAGCATATACCATAATCAACAAGACTTTGATGGAGGCTGAGGC 1320

||||||||||||||||||||||||||||||||||||||||||||||||||||||||||||

16 pass. 1261 TGTCCCTGGGTTCGGAAAAGCATATACCATAATCAACAAGACTTTGATGGAGGCTGAGGC 1320

2 pass. 1321 TCACTACAAGTCAGTCCGGACTTGGAATGAGATCGTCCCCTCAAAAGGGTGTTTAAGAGT 1380

||||||||||||||||||||||||||||||||||||||||||||||||||||||||||||

16 pass. 1321 TCACTACAAGTCAGTCCGGACTTGGAATGAGATCGTCCCCTCAAAAGGGTGTTTAAGAGT 1380

2 pass. 1381 CGAAGGGAGGTGTCATCCTCATGTAAACGGGGTATTTTTCAATGGTATAATATTAGGGCC 1440

||||||||||||||||||||||||||||||||||||||||||||||||||||||||||||

16 pass. 1381 CGAAGGGAGGTGTCATCCTCATGTAAACGGGGTATTTTTCAATGGTATAATATTAGGGCC 1440

2 pass. 1441 TGACGGCCATGTTCTAATCCCAGAGATGCAATCATCCCTCCTCCAGCAACATATGGAGTT 1500

||||||||||||||||||||||||||||||||||||||||||||||||||||||||||||

16 pass. 1441 TGACGGCCATGTTCTAATCCCAGAGATGCAATCATCCCTCCTCCAGCAACATATGGAGTT 1500

2 pass. 1501 ATTGGAATCCTCAGTTATTCCCCTGATGCACCCCCTTGCAGACCCGTCCACGGTTTTCAA 1560

||||||||||||||||||||||||||||||||||||||||||||||||||||||||||||

16 pass. 1501 ATTGGAATCCTCAGTTATTCCCCTGATGCACCCCCTTGCAGACCCGTCCACGGTTTTCAA 1560

2 pass. 1561 GGAAGGCGATGAGGCGGAGGACTTTGTAGAAGTTCACTTTCCAGATGTGCATAAAAAGGT 1620

||||||||||||||||||||||||||||||||||||||||||||||||||||||||||||

16 pass. 1561 GGAAGGCGATGAGGCGGAGGACTTTGTAGAAGTTCACTTTCCAGATGTGCATAAAAAGGT 1620

2 pass. 1621 CTCAGAGGTTGACCTGGGTCTCCCGAACTGGGGAGAGTATGTATTACTGAGTGCAGGGAC 1680

||||||||||||||||||||||||||||||||||||||||||||||||||||||||||||

16 pass. 1621 CTCAGAGGTTGACCTGGGTCTCCCGAACTGGGGAGAGTATGTATTACTGAGTGCAGGGAC 1680

2 pass. 1681 CCTGATTGCCTTGATGTTGATAATTTTCCTAATGATATGTCGTAGAAGAGTCAATAGACC 1740

||||||||||||||||||||||||||||||||||||||||||||||||||||||||||||

16 pass. 1681 CCTGATTGCCTTGATGTTGATAATTTTCCTAATGATATGTCGTAGAAGAGTCAATAGACC 1740

2 pass. 1741 AGAATCTACGCAACGCAGTCTCAGAGGGACAGAGATGAAGGTGTCGGTCACCCCCCAAAG 1800

||||||||||||||||||||||||||||||||||||||||||||||||||||||||||||

16 pass. 1741 AGAATCTACGCAACGCAGTCTCAGAGGGACAGAGATGAAGGTGTCGGTCACCCCCCAAAG 1800

2 pass. 1801 CGGGAAATTCAAATCTTCATGGGAATCATATAAAAGTGGGGATGAAGCTAGACTGTGAAG 1860

||||||||||||||||||||||||||||||||||||||||||||||||||||||||||||

16 pass. 1801 CGGGAAATTCAAATCTTCATGGGAATCATATAAAAGTGGGGATGAAGCTAGACTGTGAAG 1860

2 pass. 1861 GCTGGTCATCCTTTCGACACTTCGAGTTCCGAAGATCACCTCCCCTTTAAGTTTGCTTTA 1920

||||||||||||||||||||||||||||||||||||||||||||||||||||||||||||

16 pass. 1861 GCTGGTCATCCTTTCGACACTTCGAGTTCCGAAGATCACCTCCCCTTTAAGTTTGCTTTA 1920

2 pass. 1921 TTGGGGGGAATCTCCGGGTTCAAGAGTCCTCCTTGAACTCCATGCGACAGAGTAGATTCA 1980

||||||||||||||||||||||||||||||||||||||||||||||||||||||||||||

16 pass. 1921 TTGGGGGGAATCTCCGGGTTCAAGAGTCCTCCTTGAACTCCATGCGACAGAGTAGATTCA 1980

2 pass. 1981 AGAGTCATGAGACTCTCATTAATCCTCTCAGTTGATCAAATCAAGTCATGTAGATT 2036

||||||||||||||||||||||||||||||||||||||||||||||||||||||||

16 pass. 1981 AGAGTCATGAGACTCTCATTAATCCTCTCAGTTGATCAAATCAAGTCATGTAGATT 2036

**Supplementary Figure 1.** Comparative analysis of Glycoprotein G Gene Nucleotide Sequences of Studied Cultural Samples of Shchelkovo-51 Rabies Virus Strain at passages 2 and 16.
